# Use of local anaesthetics and adjuncts for spinal and epidural anaesthesia and analgesia at German and Austrian University Hospitals: an online survey to assess current standard practice

**DOI:** 10.1186/1471-2253-10-4

**Published:** 2010-04-17

**Authors:** Bianca M Wahlen, Norbert Roewer, Peter Kranke

**Affiliations:** 1 Staff Anaesthesiologist, University of Wuerzburg, Department of Anaesthesia and Critical Care, Wuerzburg, Germany; 2 Professor and Chair, University of Wuerzburg, Department of Anaesthesia and Critical Care, Wuerzburg, Germany; 3 Consultant, University of Wuerzburg, Department of Anaesthesia and Critical Care, Wuerzburg, Germany

## Abstract

**Background:**

The present anonymous multicenter online survey was conducted to evaluate the application of regional anaesthesia techniques as well as the used local anaesthetics and adjuncts at German and Austrian university hospitals.

**Methods:**

39 university hospitals were requested to fill in an online questionnaire, to determine the kind of regional anaesthesia and preferred drugs in urology, obstetrics and gynaecology.

**Results:**

33 hospitals responded. No regional anaesthesia is conducted in 47% of the minor gynaecological and 44% of the urological operations; plain bupivacaine 0.5% is used in 38% and 47% respectively. In transurethral resections of the prostate and bladder no regional anaesthesia is used in 3% of the responding hospitals, whereas plain bupivacaine 0.5% is used in more than 90%. Regional anaesthesia is only used in selected major gynaecological and urological operations. On the contrary to the smaller operations, the survey revealed a large variety of used drugs and mixtures. Almost 80% prefer plain bupivacaine or ropivacaine 0.5% in spinal anaesthesia in caesarean section. Similarly to the use of drugs in major urological and gynaecological operations a wide range of drugs and adjuncts is used in epidural anaesthesia in caesarean section and spontaneous delivery.

**Conclusions:**

Our results indicate a certain agreement in short operations in spinal anaesthesia. By contrast, a large variety concerning the anaesthesiological approach in larger operations as well as in epidural analgesia in obstetrics could be revealed, the causes of which are assumed to be primarily rooted in particular departmental structures.

## Background

Regional anaesthesia as an alternative to general anaesthesia or to supplement general anaesthesia has become a popular procedure in clinical anaesthesiology. The development of different local anaesthetics and various techniques in regional anaesthesia had been boosted by the growing interest in regional anaesthesia due to its effective pain relief without compromising the patient's consciousness and improved patient comfort. Furthermore, it has been influenced by the implementation of perioperative anaesthesia standards and the increasing awareness among healthcare professionals that postoperative analgesia plays an important part in the reconvalescence of patients. In addition to speed up perioperative process organization, e.g. in patients undergoing "Fast-track surgery" [1], regional anaesthesia in dedicated settings offers profound clinical advantages, e.g. reduced perioperative morbidity [2-4].

Since lumbar and especially thoracic epidural anaesthesia as well as the technique of a combined spinal-epidural-anaesthesia (CSE) have found their way into clinical anaesthesia, there is an ongoing discussion about which technique to favour in which clinical setting.

In most of the academic hospitals, scientific evidence is translated into more or less fixed clinical guidelines regarding the method of regional anaesthesia for a specific procedure. These guidelines or standard operating procedures (SOP) usually not only cover the technique itself, but also the substances to be used at first instance. However, clinical practice and thus standard operating procedures do not necessarily reflect pure scientific evidence; therefore there might be a gap between scientific evidence and current clinical standards.

In addition to the use of different local anaesthetics and regional anaesthesia procedures, merging of local anaesthetics with adjuvants gained widespread popularity due to the belief, that the addition of various opioids[5,6] or other components, e.g. clonidine[7], allows the reduction of the amount of local anaesthetic and thus the incidence of side effects[8,9]. Despite the plethora of various substances, concentrations and molecules used as adjuvants for regional anaesthesia, there is an ongoing debate whether this practice really adds clinical benefit or just complicates the procedures and introduces risks for medication error[10,11]. Furthermore, there is relative paucity as far as the rationale for specific practice patterns, i.e. combination of local anaesthetic and its concentration in conjunction with an adjuvant, in distinct clinical settings is concerned.

Hence, the present anonymous online survey was conducted to evaluate the application of regional anaesthesia techniques as well as the primarily used local anaesthetics and adjuncts in urology and gynaecology, as reflected by the local standard operating procedures, at German and Austrian university hospitals.

## Methods

The online survey was conducted between June and August 2007. As the majority of German speaking anaesthesiologists spend at least one part of their specialist training at university hospitals and therefore are reflected by the approach of university hospitals, we contacted the 36 German and 3 Austrian university affiliated European anaesthesia departments via e-mail.

The e-mail contained a link (URL) that directed the recipient to an online questionnaire (the online questionnaire has been reconstructed in English for demonstration purpose; http://www.notyetinc.de/msla/). The final questionnaire was developed following a survey among a focus group of anaesthesiologists interested in the topic. The initial version was tested in a pilot phase among local anaesthetists in order to ensure readability and eliminate questions that may evoke erroneous answers. With respect to the fact that at German speaking university hospitals at least one person is responsible for the internal guidelines, the initial mail was directed to the department chairs indicating the purpose and method of the survey asking for forwarding the request to the responsible consultant for regional anaesthesia. In the questionnaire participants were requested to select one of the clinical subspecialties: urology or gynaecology. In these sub-menus participants were able to choose the relevant surgical procedures that are performed in the University's Department of Urology, Departments of Gynaecology and in Obstetrics. The provided classifications for the urological procedures were "urological procedures with duration shorter than two hours", "transurethral resection of the prostate (TURP)", "transurethral resection of the bladder (TURB)", "larger urological operations with duration longer than two hours". The corresponding classifications for gynaecological procedures and obstetrical procedures were "gynaecological operations with duration shorter than two hours", "larger gynaecological operations with duration longer than two hours", "Caesarean section", "labour pain for spontaneous vaginal delivery". For each of these procedures, substances ("articaine", "bupivacaine", "etidocaine", "lidocaine", "mepivacaine", "prilocaine", "procaine", "ropivacaine", "tetracaine") and techniques ("spinal anaesthesia", "epidural anaesthesia", "combined spinal epidural (CSE)") could be chosen out of a pre-determined drop down list. Adjuvants, e.g. sodium chloride 0.9%, morphine, fentanyl, sufentanil, clonidine, could be indicated using free-text fields. Hospitals, which did not complete the form until September 2007 were once contacted via telephone and/or e-mail and asked to complete the survey. The entries were online transferred to and stored in a database using "Microsoft ACCESS" and subsequently described and analyzed. For further analysis of the results a ranking list was constructed to gain an overview of the standards and the most often used regional anaesthesia techniques and local anaesthetic solutions as well as adjuvants at German and Austrian university hospitals. Data are presented as number (n) and percentage (%).

## Results

33 out of 39 (85%) German and Austrian university hospitals responded.

### A. Urology

#### 1. Urological operations < 2 hours

**Figure 1:**
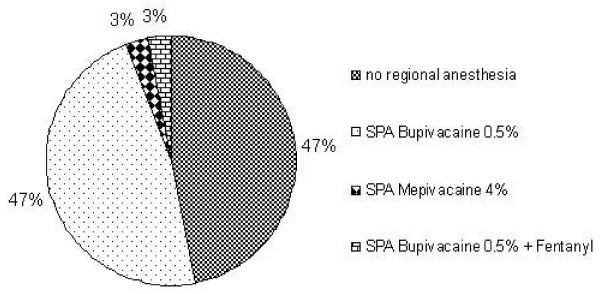
**Urological operations < 2 hours (n = 33)**.

In short urological procedures almost half of the respondent departments stated that they do not use regional anaesthesia techniques. Only a small number of participants use short-acting local anaesthetics (i.e. mepivacaine), while the majority, in case of performing central neuraxial regional anaesthesia, prefer a spinal anaesthesia with bupivacaine 0.5% (Figure 1).

#### 2. Transurethral resection of the bladder (TURB)/Transurethral resection of the prostate (TURP)

**Figure 2:**
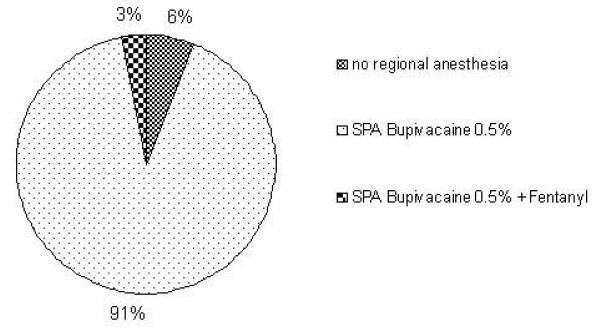
**Transurethral resection of the bladder (TURB)/Transurethral resection of the prostate (TURP) (n = 33)**.

More than 90% of the responding hospitals regularly use bupivacaine 0.5% without any adjuncts. Only 3% stated that they do not use regional anaesthesia techniques in these types of surgery at all. Adding fentanyl to bupivacaine was the only mentioned adjunct for transurethral resections (Figure 2).

#### 3. Urological operations > 2 hours

For longer urological procedures, a quarter of the respondents stated that they do not use regional anaesthesia techniques in these types of surgery. In contrast to transurethral resections, spinal anaesthesia with bupivacaine 0.5% does not play a major role (6%). Epidural analgesia in conjunction with a general anaesthetic is quite popular in longer lasting urological procedures. Ropivacaine (range of the concentrations used: 0.16 - 0.75%) is slightly more frequently used (~40%) than bupivacaine (range of the concentrations used: 0.25%-0.5%), which is used by approximately 30% of the respondents as first-line drug for epidural analgesia. Sufentanil is the only mentioned adjuvant drug for epidural administration either in conjunction with bupivacaine or ropivacaine (Table 1). Epidural analgesia is supplemented routinely with sufentanil in approximately 75% of the respondent departments.

**Table 1:** Preferred use of regional anaesthesia technique for large urological operations.

Technique	Local anaesthetic	Concentration of the local anaesthetic	Adjuvant	Distribution (n = 33)
no regional anaesthesia			n.a.	8 (25%)
Epidural anaesthesia	Bupivacaine	0.5%	none	4 (12%)
Epidural anaesthesia	Bupivacaine	0.25%	Sufentanil	3 (9%)
Epidural anaesthesia	Ropivacaine	0.2%	Sufentanil	3 (9%)
Epidural anaesthesia	Bupivacaine	0.5%	Sufentanil	3 (9%)
Epidural anaesthesia	Ropivacaine	0.375%	Sufentanil	3 (9%)
Epidural anaesthesia	Ropivacaine	0.5%	Sufentanil	3 (9%)
Spinal anaesthesia	Bupivacaine	0.5%	Sufentanil	2 (6%)
Epidural anaesthesia	Ropivacaine	0.16%	Sufentanil	1 (3%)
Epidural anaesthesia	Ropivacaine	0.5%	none	1 (3%)
Epidural anaesthesia	Ropivacaine	0.75%	none	1 (3%)
Epidural anaesthesia	Ropivacaine	0.75%	Sufentanil	1 (3%)

Table shows type of regional anaesthesia, local anaesthetic, concentration of the local anaesthetic as well as preferred adjuvants (e.g. opioids). Distribution is given in number (proportion).

### B. Gynaecology

#### 1. Gynaecological operations < 2 hours

**Figure 3:**
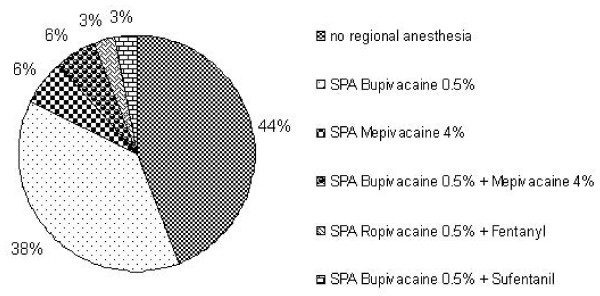
**Gynaecological operations < 2 hours (n = 33)**.

More than 40% of the departments do not routinely use regional anaesthesia techniques in gynaecological operations shorter than two hours. For the remaining departments, spinal anaesthesia with various local anaesthetics is the preferred choice of regional anaesthesia. Of them, almost 40% prefer plain bupivacaine 0.5%. Other substances or adjuvants (sufentanil or fentanyl), or even combinations of short and long-acting local anaesthetics are used, but play a negligible role (Figure 3).

#### 2. Large gynaecological operations

For longer gynaecological procedures, almost half of the respondents stated that they do not use regional anaesthesia techniques in these types of surgery. Spinal anaesthesia was not mentioned at all. Epidural analgesia in conjunction with a general anaesthetic is quite popular in longer lasting gynaecological procedures.

Ropivacaine (range of the concentrations used: 0.16 - 0.75%) is approximately as often mentioned (~30%) as bupivacaine (range of the concentrations used: 0.25%-0.5%) being the drug of first choice for epidural analgesia. Sufentanil is the only mentioned adjuvant drug for epidural administration either in conjunction with bupivacaine or ropivacaine. The overwhelming fraction of respondent departments supplements an epidural analgesia with sufentanil (Table 2).

**Table 2:** Preferred use of regional anaesthesia technique for large gynaecological operations.

Technique	Local anaesthetic	Concentration of the local anaesthetic	Adjuvant	Distribution (n = 33)
no regional anaesthesia			n.a.	14 (43%)
Epidural anaesthesia	Bupivacaine	0.5%	None	4 (12%)
Epidural anaesthesia	Ropivacaine	0.2%	None	3 (9%)
Epidural anaesthesia	Bupivacaine	0.5%	Sufentanil	3 (9%)
Epidural anaesthesia	Ropivacaine	0.2%	Sufentanil	3 (9%)
Epidural anaesthesia	Bupivacaine	0.25%	Sufentanil	2 (6%)
Epidural anaesthesia	Ropivacaine	0.16%	Sufentanil	1 (3%)
Epidural anaesthesia	Ropivacaine	0.5%	Sufentanil	1 (3%)
Epidural anaesthesia	Ropivacaine	0.375%	Sufentanil	1 (3%)
Epidural anaesthesia	Ropivacaine	0.75%	Sufentanil	1 (3%)

Table shows type of regional anaesthesia, local anaesthetic, concentration of the local anaesthetic as well as preferred adjuvants (e.g. opioids). Distribution is given in number (proportion).

#### 3. Spinal anaesthesia for caesarean section

**Figure 4:**
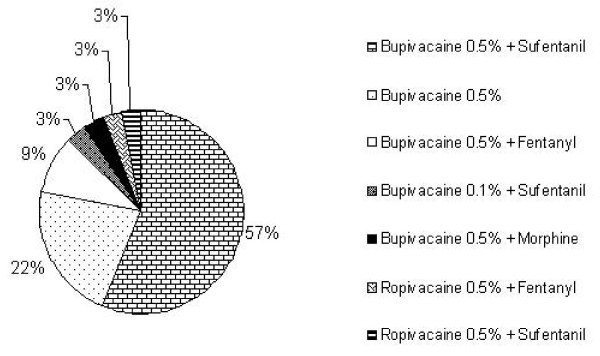
**Spinal anaesthesia for caesarean section (n = 33)**.

Almost 60% of the responding hospitals use a combination of bupivacaine 0.5% with sufentanil, whereas slightly more than 20% prefer plain bupivacaine 0.5% alone. Apart from bupivacaine some departments also use ropivacaine as standard local anaesthetic. Fentanyl and morphine are used as adjuvants apart from sufentanil only in a small fraction of respondent departments (Figure 4).

#### 4. Epidural anaesthesia for caesarean section

The vast majority of the participating hospitals prefer plain ropivacaine 0.75%, followed by ropivacaine and bupivacaine 0.5%, each supplemented with Sufentanil. Other local anaesthetics and adjuvants (fentanyl) do not play an important role as first-line drugs (Table 3).

**Table 3:** Preferred local anaesthetic, concentration of the local anaesthetic as well as preferred adjuvants for epidural anaesthesia in caesarean section.

Local Anaesthetic	Concentration of the local anaesthetic	Adjuvant	Distribution (n = 33)
Ropivacaine	0.75%	none	12 (37%)
Ropivacaine	0.5%	Sufentanil	9 (27%)
Bupivacaine	0.5%	Sufentanil	6 (18%)
Lidocaine	2%	none	1 (3%)
Ropivacaine	0.5%	none	1 (3%)
Ropivacaine	1%	Sufentanil	1 (3%)
Bupivacaine	0.125%	none	1 (3%)
Ropivacaine	0.5%	Sufentanil	1 (3%)
Bupivacaine	0.25%	Fentanyl	1 (3%)

Distribution is given in number (proportion).

#### 5. Epidural analgesia in spontaneous delivery

Almost 40% of the responding departments use ropivacaine 0.2% with sufentanil as adjunct, followed by ropivacaine 0.1% with sufentanil, bupivacaine 0.125% with sufentanil, plain ropivacaine 0.2% without adjuvants and bupivacaine 0.1% with sufentanil. The observed diversity of the used concentrations was considerable. However, some concentrations only play a niche role (Table 4).

**Table 4:** Preferred local anaesthetic, concentration of the local anaesthetic as well as preferred adjuvants for epidural anaesthesia in spontaneous delivery.

Local anaesthetic	Concentration of the local anaesthetic	Adjuvant	Distribution (n = 33)
Ropivacaine	0.2%	Sufentanil	12 (37%)
Ropivacaine	0.1%	Sufentanil	4 (12%)
Bupivacaine	0.125%	Sufentanil	3 (9%)
Ropivacaine	0.2%	None	3 (9%)
Bupivacaine	0.1%	Sufentanil	2 (6%)
no regional anaesthesia		n.a.	1 (3%)
Bupivacaine	0.13%	Sufentanil	1 (3%)
Ropivacaine	0.075%	Sufentanil	1 (3%)
Ropivacaine	0.1%	None	1 (3%)
Ropivacaine	0.16%	Sufentanil	1 (3%)
Ropivacaine	0.175%	Sufentanil	1 (3%)
Ropivacaine	0.18%	Sufentanil	1 (3%)
Ropivacaine	0.25%	Sufentanil	1 (3%)
Ropivacaine	0.2%	Fentanyl	1 (3%)

Distribution is given in number (proportion).

## Discussion

The present anonymous online survey at German and Austrian university hospitals was conducted to evaluate the application of regional anaesthesia techniques and preferred local anaesthetics with concentrations used, including possible adjuvants, for common operations in urology, gynaecology and obstetrics.

Our results suggest that there exists some consensus regarding the techniques of regional anaesthesia that are considered useful for specific procedures with a pre-defined duration in urology, gynaecology and obstetrics among German and Austrian University Hospitals. In contrast to the observed agreement as far as a common standard approach for regional anaesthesia techniques is concerned, the choice of the local anaesthetics, concentrations of local anaesthetics used and/or adjuvants differs to a large extent in the university departments included in this survey.

The present poll revealed that spinal anaesthesia is quite popular in "minor" urological as well as gynaecological operations. Plain bupivacaine 0.5% was the favourite substance in these short procedures. Considering the fact that the majority of minor gynaecological as well as urological operations are performed on an outpatient basis, which in turn means, that procedural times are quite short and less pain sensation occurs postoperatively, it is interesting to note that the replying clinics do not use short acting substances regularly, e.g. prilocaine. The mixture of bupivacaine 0.5% with mepivacaine 4%, which was used by a small fraction of departments has a slight advantage over plain bupivacaine 0.5% concerning the onset time of analgesia, whereas concerning the maximal level of analgesia or the duration of sensory or motor blockade both alternatives seem to be equipotent[12]. The infrequent use of short-acting substances may, at least in part, be explained by the fact that only university departments with the risk of longer intraoperative process times were contacted.

The frequent use of plain bupivacaine as local anaesthetic in urology and gynaecology is striking, since especially in those subspecialties, a high percentage of the patients are older aged with a considerable prevalence of cardio-circulatory disorders. In those patients the need for cardiovascular stability may be extremely important and a well known side effect of large doses of local anaesthetics is the negative influence on cardiocirculatory parameters, mainly vasodilatation due to sympathicolysis[13]. The common knowledge that the addition of opioids allows a reduction of local anaesthetics and consequently leads to a lower incidence of cardiovascular side effects[8,9] is only partly reflected by the results of this survey, where the overwhelming majority uses plain bupivacaine in short gynaecological and urological operations.

In the past years, the mixture of local anaesthetics with adjuncts, such as opioids, has gained widespread popularity. Sufentanil is the overwhelming choice for epidural analgesia. On the contrary, the addition of opioids to spinal regional anaesthesia, apart from patients undergoing a caesarean section, does not play a major role.

Concerning the anaesthesiological management of patients undergoing larger gynaecological (e.g. abdominal hysterectomies) or larger urological (e.g. radical prostatectomies) procedures, the present study revealed a large variety of regional anaesthesia techniques used as the first-line perioperative analgesia technique.

Especially epidural analgesia is used in the respondent departments on a regular basis. Bupivacaine or ropivacaine are clearly the drugs of choice in various concentrations with or without the use of adjuncts.

There is little doubt that in major surgical procedures, such as retropubic radical prostatectomy, nephrectomy, abdominal hysterectomy or Wertheim-Meigs procedures, epidural analgesia is an excellent approach to relieve severe dynamic perioperative pain and it has been shown, that there is a reduced likelihood of developing post-operative respiratory tract infections[14] and cardiac ischemic events[3] in patients, who are at high risk for those complications[15]. On the other hand, the economic costs of such services are considerable[16,17] and overall cost-efficiency has to take into account the fraction of patients not consenting to the procedures, patients with known contraindications for the procedure and early or late failures/losses of the epidural catheter[18]. Therefore, in departments, where the establishment of an acute pain service is not feasible, but the necessity of a sufficient perioperative pain management is acknowledged, a balance between resource allocation and incremental benefits of the provided analgesia procedure need to be established. Therefore holistic process evaluations may have greater impact on the current standard operating procedures regarding neuraxial analgesia than some scientific evidence in selected patient populations.

With respect to obstetrics remarkable distinctions between different countries and a constant change concerning the use of various substances during the past decade, as reflected by the results of national as well as international surveys, could be observed.

Previous postal surveys in the UK conducted by Burnstein [19] as well as Jones [20] showed that in the UK bupivacaine is the most often used local anaesthetic. Furthermore, Burnstein was also able to demonstrate an increase in the use of opioids in the United Kingdom since 1991 [19].

In contrary to the surveys conducted by Jones and Burnstein our results indicate a complete different approach to epidural analgesia in labour concerning the used substances. Only 18% of the Austrian and German hospitals use bupivacaine at all. The vast majority prefers ropivacaine in various concentrations. 85% of the German speaking university hospitals add opioids as adjunct. The vast majority (97%) uses sufentanil and only 3% prefer fentanyl. In contrast, Burnstein was able to demonstrate that in the United Kingdom fentanyl is the most commonly employed opioid and to a smaller extend alfentanil and diamorphine are used. The fact, that fentanyl is obviously the most popular opioid in European, English speaking countries, is confirmed by another postal survey conducted by Carson and colleagues in 1996. They were able to display that in 41% of the cases opioids are used in labour, with fentanyl given by a bolus dose being the commonest drug and method of administration[21].

Further, not only in contrast to other European countries a remarkable change concerning the used local anaesthetics that occurred within the last twelve years could be revealed.

An online survey by Stamer and colleagues conducted in 1996 showed that 80% of the contacted German units use ropivacaine 0.75% for epidural anaesthesia in patients undergoing caesarean section; 62% ad an opioid as adjunct. Of these, 56.5% prefer sufentanil, 5% fentanyl and 1.3% morphine[22].

During the last decade the absolute number of hospitals using 0.75% solutions has decreased by more than 50%, tending towards 0.5% solutions, whereas the use of opioids as adjuvants for epidural anaesthesia remained almost stable.

In 1996, the preferred local anaesthetic for spinal anaesthesia in caesarean section proved to be bupivacaine in 85.1%. Mepivacaine was used in 5.1% of the cases.

More than one third of the participants of this survey combined local anaesthetics with intrathecal opioids. Sufentanil was the most popular opioid; fentanyl and morphine were only used by a very small number of units.

Comparing the frequency of the use of substances in spinal anaesthesia for caesarean section between 1996 and 2007 reveals a further increase in the use of bupivacaine. The combination of local anaesthetics with opioids skyrocketed in the past decade, with more than 2/3 of the units using such a mixture. The most popular substance is clearly intrathecal sufentanil.

A weakness of the present survey is clearly the fact that only university departments were contacted. However, we sought to determine how especially those institutions weigh the current evidence regarding the use of regional anaesthesia techniques and how academic departments transfer this knowledge to standard operating procedures.

Further, we have not presented much insight regarding specific procedures or reasons for or against a specific technique with one or the other surgical procedure. Since clinical trial investigating different methods of regional anaesthesia are usually restricted to a dedicated patient population and focus on a specific outcome, it may be speculated whether benchmark projects offer better overall insights regarding the superiority of one method or local anaesthetic over the other[23].

### Limitations of the study

In focussing mainly on the preferred technique and used local anaesthetics we were unable to provide information concerning the distribution between general anaesthesia and the given types of regional anaesthesia or between emergency or scheduled operations, e.g. caesarean section. This remains to be evaluated in further surveys.

## Conclusions

Our results indicate a certain agreement among the responding university hospitals concerning the preferred type of regional anaesthesia, especially in minor gynaecological and urological operations. By contrast, a large variety concerning the anaesthesiological approach (regional anaesthesia technique) as well as the used drugs or adjuvants in larger operations could be revealed. The observed diversity was even more pronounced as far as concentrations used and adjuvants to local anaesthetics are concerned. Causal relationships for preferring one or the other approach were not investigated. However, the observed huge variety can hardly be explained by sound scientific evidence and we assume that the underlying causes are rooted primarily in particular departmental structures, historical developments and personal experiences and preferences.

## Competing interests

The authors declare that they have no competing interests.

## Authors' contributions

BMW designed the web page for the online survey, participated in the design of the study, in the sequence alignment and drafted the manuscript. PK participated in the sequence alignment, performed the statistical analysis and drafted the manuscript. NR participated in the design of the study, in the sequence alignment and drafted the manuscript. All authors have read and approved the manuscript.

## Pre-publication history

The pre-publication history for this paper can be accessed here:

http://www.biomedcentral.com/1471-2253/10/4/prepub
